# Whole-Genome Sequencing Reveals the Progress of Genetic Breeding in *Eriocheir sinensis*

**DOI:** 10.3390/ani15010077

**Published:** 2025-01-01

**Authors:** Xuanpeng Wang, Gaowei Zhang, Dandan Gao, Yongchun Ge, Yongxu Cheng, Xinhai Wang

**Affiliations:** 1 Suqian Institute of Agricultural Sciences, Jiangsu Academy of Agricultural Sciences, Suqian 223800, China; 15805243017@163.com (X.W.); feifeidou1@sina.com (G.Z.); 18135777653@163.com (D.G.); 2 Suqian King Crab Industry Research Institute, Suqian 223800, China; geyongchun123@163.com; 3 National Demonstration Centre for Experimental Fisheries Science Education, Shanghai Ocean University, Shanghai 201306, China

**Keywords:** *Eriocheir sinensis*, genetic improvement, whole-genome sequencing, genetic diversity, genetic distance

## Abstract

**Simple Summary:**

A new variety of *E*. *sinensis* urgently needs to be produced though genetic breeding. Our research group collected crabs from Yixing and Suqian populations and aimed to produce a new variety of *E*. *sinensis* through mass selection. The progress of the genetic breeding of *E. sinensis* was evaluated through whole-genome sequencing. Generally, individuals from the G1 generation showed better growth performance than those from the G0 generation. In addition, crabs from the G0 and G1 generations shared close genetic backgrounds, and the G0 and G1 generations showed a high level of genetic diversity and a relatively stable genetic structure.

**Abstract:**

*Eriocheir sinensis* is an important and popular crustacean species in China, producing huge economic benefits. Large individuals of *E. sinensis* are preferred due to market demand. The long-term goal of our research group is to produce a new variety of *E. sinensis* with better growth performance and stronger abilities to resist environmental changes through mass selection. The present study aimed to evaluate the progress of the genetic breeding of *E. sinensis* by analyzing the genetic diversity and genetic distance between the basic breeding population (pooled population of Suqian and Yixing, G0) and generation 1 (G1) using whole-genome sequencing (WGS). The growth traits, including body weight, shell length, shell width, and third appendage length, in the G1 generation increased by 8.3%, 7.9%, 9.6%, and 9.3%, respectively, compared with those of the G0 generation, indicating that the G1 generation showed better growth performance. A total of 372,448,393 high-quality single nucleotide polymorphisms (SNPs) were detected in 40 *E. sinensis* individuals, with an average of 9,331,209.83 SNPs. The fixation index values were 0.007 between the Yixing and Suqian populations and 0.015 between the G0 generation and G1 generation, indicating a close genetic background between these groups, especially when considered in combination with the phylogenetic tree and principal component analysis. All of these data suggest that genetic information was stably inherited by the G1 generation, with no introduction of foreign genetic information during the genetic breeding process. In addition, the genetic diversity analysis revealed that the G0 and G1 generations showed a high level of genetic diversity and a relatively stable genetic structure. The present study evaluated the recent progress of the genetic improvement of *E. sinensis* by our research group, providing valuable evidence for further genetic improvement in this species. Future studies will be performed to select growth-related SNPs and genes through genome-wide association studies.

## 1. Introduction

*Eriocheir sinensis* is an economically important crustacean species in China. It is widely distributed across all of China, including the Yangtze River, Liaohe River, Yellow River, and Pearl River [1]. This species is a popular aquatic animal in China because of its desirable taste and rich nutritional content [2,3]. *E. sinensis* belongs to a migratory crustacean group that prefers to inhabit caves along rivers and lakes in freshwater environments. However, it needs to migrate to brackish water resources for spawning [4]. *E. sinensis* has several developmental stages, including the zygote, zoea, megalopa, juvenile crab, young crab, and adult crab stages [5]. *E. sinensis* needs to molt several times from the zoea stage to the adult stage, promoting its growth and development [6,7]. Many factors have been identified to affect the growth of *E. sinensis*, including shelters [8], environmental temperature [9], background color [10], and food sources [11,12]. The annual production of *E. sinensis* reached 808,558 tons in 2023, producing huge economic benefits. The main aquaculture regions include Jiangsu Province, Hubei Province, and Anhui Province, where annual production totaled more than 100,000 tons. Jiangsu Province is the most important aquaculture region for *E. sinensis*, accounting for about 50% of the total annual production and economic benefits [13].

The intensive aquaculture practices of *E. sinensis* have led to the degradation of germplasm resources, resulting in poor-quality seedlings, smaller adult crabs, lower yields, increased sensitivity to environmental changes, and a higher proportion of early maturity, restricting the sustainable development of the *E. sinensis* industry. The possible reasons for this include inbreeding and reverse selection [14]. Genetic breeding through artificial selection provides numerous superior breeding varieties for aquatic animals, which dramatically promote the production output, playing essential roles in the maintenance of sustainable development for the aquaculture industry [15]. Artificial selection has been performed in the *E. sinensis* industry in order to provide high-quality seedlings for market demand. In recent decades, many new varieties of *E. sinensis* have been selected and produced through hybrid selection, mass selection, and family selection based on their growth performance. These varieties have been approved by the Ministry of Agriculture and Rural Affairs of China. However, the seedlings of new varieties of *E. sinensis* are insufficient for the market’s needs. In addition, the new varieties of *E. sinensis* are sensitive to various environmental changes and pollution, leading to high mortality during aquaculture programs. Environmental changes and pollution include high temperatures, heavy metal ions [16], pesticides [17], and nitrites [18]. Thus, it is still necessary to perform genetic improvement in this species in order to obtain new varieties with better growth performance and stronger abilities to resist environmental stress. 

Whole-genome sequencing (WGS) is a useful and essential method for the analysis of genetic diversity and adaptive evolutionary traits in various animals, including fish [19], pigs [20], sheep [21], and chickens [22]. WGS refers to the systematic analysis of individual or population differences in a species by sequencing the entire genome sequence of different individuals of a species for which the whole-genome sequence has already been obtained [23]. Both natural and artificial selection leave selection signatures on a genome, affecting the genome sequences of animal offspring. The selection signatures can be observed as an increase in chromosome linkage disequilibrium at the selection site or a decrease in genetic diversity. The selection signatures can be identified by performing WGS, and the population structure and evolutionary history of populations can be analyzed [24]. The cost of next-generation sequencing (NGS) technology has been dramatically reduced because of its fast and continuous development in recent decades. Thus, WGS based on NGS has been considered and widely used as a comprehensive, cost-effective, and rapid tool for the analysis of genetic diversity in aquatic animals [25,26,27].

Genetic diversity is an important concept in evolutionary biology and molecular ecology, playing an essential role in the analysis of the complexity of organisms, the adaptation of species to diverse environmental conditions, and the restoration of ecosystems [28,29]. It is also a crucial theoretical foundation for genetic improvement. Analyses of genetic diversity promote studies of evolutionary history and future directions in a species or populations, and they provide valuable evidence for the conservation of genetic resources, the sustainable utilization of wild resources, and the development of biological resources [30]. A previous study predicted that conventional breeding methods may lead to an 8–12% increase with every generation during the artificial selection of fish and shrimp [31]. However, recent studies revealed that aquatic animals have only obtained limited genetic improvement or domestication [32]. Therefore, fully utilizing the wide genetic diversity in wild populations plays an essential role in the improvement of aquaculture productivity during breeding programs [31]. Thus, a comprehensive understanding of the genetic structure of different groups is essential prior to the rational and optimized use of genetic diversity resources from wild populations.

The wild *E. sinensis* populations in the Yangtze River have advantages over other wild *E. sinensis* populations, including a larger body size and specific taste [33]. However, several possible effects have led to a decrease in the wild population of this species in the Yangtze River in the last few decades, including dam construction, overfishing, and water pollution. In addition, the germplasm resources of *E. sinensis* in the Yangtze River were observed to be degraded in recent years, especially those of size and taste. Experts provided two possible reasons for this degradation. The first is that crab populations from Yangtze River could interbreed with populations from the Liaohe River in the northeast of China and the Oujiang River in the south of China, leading to the degradation and dilution of this preferred germplasm. The second viewpoint is that crab populations could not interbreed between these three large rivers due to the difference in the spawning time [34]. In general, most experts supported the former view, resulting in the degradation of germplasm resources in the Yangtze River, resulting in a small size and early maturity. Thus, the reasonable utilization of wild crab populations from the Yangtze River for genetic improvement is preferred for the *E. sinensis* industry. Previous studies have been performed to evaluate the germplasm resources and analyze the genetic diversity and population structure of *E. sinensis* [33,35,36,37]. 

The present study collected wild *E. sinensis* populations from Suqian and Yixing in Jiangsu Province and aimed to perform genetic improvement in this species through mass selection. We analyzed the genetic diversity in the G0 and G1 generations and evaluated the genetic distance between the G0 and G1 generations after performing the artificial selection in *E. sinensis*. The present study will provide valuable evidence for the evaluation of the progress of genetic improvement in *E. sinensis*.

## 2. Materials and Methods

### 2.1. Tissue Collection

**Figure 1:**
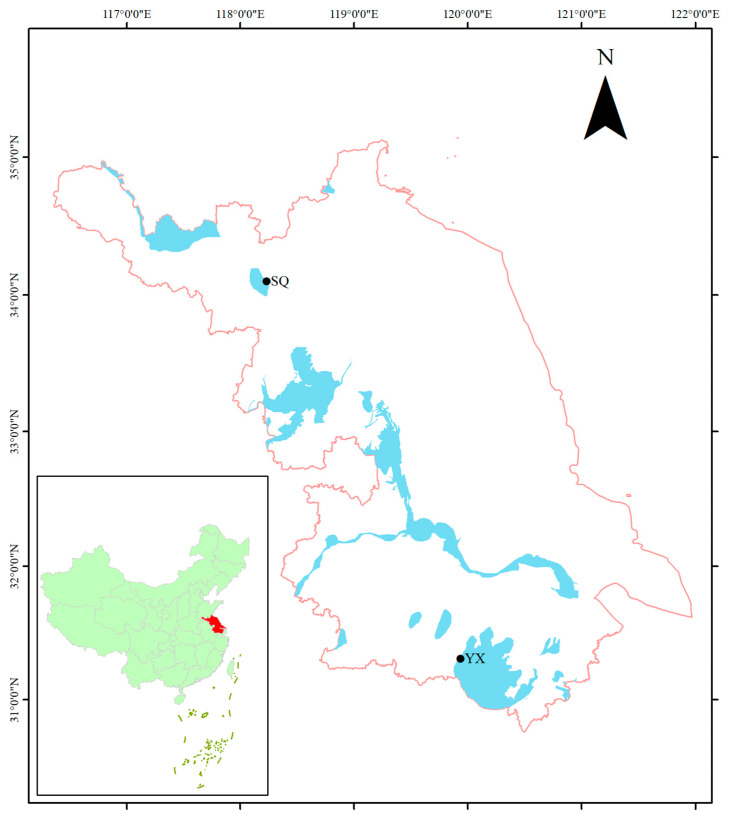
Sampling map of *E. sinensis* in the present study. SQ indicates the Suqian population. YX indicates the Yixing population.

Wild *E. sinensis* populations were collected from Suqian (Lumahu Lake, 117°56′19″–119°10′ E, 33°8′–34°25′ N) and Yixing (Taihu Lake, 119°31′–120°03′ E, 31°07′–31°37′ N) in Jiangsu Province (Figure 1). A total of 3000 individuals with good growth performance were selected to form a basic breeding population, which was labeled as the G0 generation. The G0 population was cultured and bred in Jinyang Aquatic Seed Farm (Sheyang, Jiangsu Province, 120°15′36″ E, 33°46′48″ N), which was close to the estuary. The water resource used for crab breeding was collected directly from the estuary. The crabs were maintained in water conditions for breeding with a saline concentration of 15–21‰, pH of 7.0–8.5, dissolved oxygen of >6 mg/L, and water temperature of 8–12 °C. A total of 20 individuals (10 males and 10 females) were randomly selected from the G0 generation for the analysis of genetic diversity and genetic distance, and they were labeled from G0-01 to G0-20 (G0-01 to G0-10 were from the Suqian population, and G0-11 to G0-20 were from the Yixing population). A total of 100 individuals (50 males and 50 females) from the G0 generation were randomly selected to measure their growth traits, including their weight, shell length, shell width, and third appendage length. Growth traits were measured according to the criteria of GB/T 19783-2005 [38].

All of these 3000 wild *E. sinensis* individuals were used as parents to breed together, and their offspring were labeled as the G1 generation. A total of 20 individuals (10 males and 10 females) were randomly selected from the G1 generation for the analysis of genetic diversity and genetic distance, and they were labeled from G1-01 to G1-20. A total of 100 individuals (50 males and 50 females) from the G1 generation were randomly selected; their growth traits were measured, and they were consistent with those of the G0 generation. Muscle samples were collected from each individual and immediately frozen in liquid nitrogen in order to prevent DNA degradation. All of the muscle tissues were stored in a refrigerator at –80 °C until DNA extraction. SPSS Statistics 23.0 (IBM, Armonk, NY, USA) was used to conduct statistical analyses of the growth traits of the G0 and G1 generations by using a paired t-test. A *p*-value of <0.05 indicated a significant difference.

### 2.2. DNA Extraction, Whole-Genome Resequencing, and Variation Calling

Deoxyribonucleic acid (DNA) was extracted from each muscle sample by using a TIANamp Marine Animals DNA Kit (Tiangen, Beijing, China) according to the manufacturer’s instructions. DNA quality was determined using both 1.2% agarose gel electrophoresis and an Agilent 4200 Bioanalyzer (Agilent Technologies, Santa Clara, CA, USA). In addition, the DNA concentration was measured by using an ultraviolet spectrophotometer (Eppendorf, Hamburg, Germany).

The WGS in the present study was based on the PE libraries (2 × 150 bp) and was performed on the Illumina NovaSeq^TM^ sequencing platform (Majorbio Biotech, Shanghai, China) according to the manufacturer’s instructions. Fastp (version 0.20.0) with the default parameters was used to perform quality control of the raw sequencing data [39]. Raw reads with adaptors and low-quality bases were filtered out, and clean reads were obtained. The clean reads were then aligned to the *E. sinensis* reference genome from NCBI (https://www.ncbi.nlm.nih.gov/datasets/genome/GCF_024679095.1/, accessed on 5 September 2022) by using BWA-MEM (version 0.4.17) [40]. The mapped results were finally converted into BAM format, and SAMtools (version 1.11) was then employed to sort the aligned sequences [41]. Picard (version 3.0) (https://github.com/broadinstitute/picard, accessed on 11 November 2022) was employed to remove the replicates. GATK (version 4.5.0.0) was used to screen the SNP sites and indels [42]. PLINK (version 1.9 beta 7.7) was employed to convert the resulting file format, and the low-quality SNPs were filtered out using the criterion of a minor allele frequency (MAF) of <0.05 and a call rate of <70% [43]. The effects of the filtered SNPs were predicted by using snpEff v4.3 based on the genome annotation file [44].

### 2.3. Phylogenetic and Linkage Disequilibrium (LD) Analysis

Phylogenetic analysis was performed based on the filtered single nucleotide polymorphism markers (SNPs). The maximum likelihood and the neighbor-joining tree were constructed by using IQ-TREE2 with the GTR + I + G4 model and 1000 bootstraps [45], and the FastTree2 software (v2.1.11) with the -gtr-gamma model and 1000 bootstraps [46], respectively. PLINK was employed to perform a principal component analysis (PCA) of whole-genome SNPs in order to measure the genetic relationships among samples [43]. PopLDdecay (v3.40) was used to evaluate the LD decay across the genome by calculating the squared correlation (*r*^2^) between any two loci based on the resequencing data [47]. The average *r*^2^ value was calculated for pairwise SNPs in a 500 kb region and averaged across the whole genome.

### 2.4. Genetic Diversity and Genetic Distance Analysis

Genetic diversity analysis in a population or an organism plays an essential role in the status of biological resources and the protection and management of wildlife. In the present study, the genetic diversities in a generation were evaluated using various parameters, including the expected heterozygosity (*He*), observed heterozygosity (*Ho*), nucleotide diversity (*Pi*), and polymorphism information content (*PIC*), based on the high-quality SNPs generated using WGS with a minor allele frequency (MAF) of >0.01. The value of *He* and *Ho* for each SNP site in each generation was calculated using the PLINK software [43]. The Anderson formula was used to evaluate the *PIC* value of each SNP site in each generation [48]. VCFtools was used to estimate the nucleotide diversity in each generation, which was measured using the *Pi* value with a 10 kb window and 10 kb sliding steps. 

Population differentiation between the G0 and G1 generations was evaluated by using the fixation index (*Fst*). The value of *Fst* ranges from 0 to 1, where a value of 0 indicates no differentiation, and a value of 1 suggests complete differentiation. PLINK (version 1.9 beta 7.7) was used to evaluate the *Fst* value between the Yixing population and Suqian population, as well as between the G0 and G1 generations.

## 3. Results

### 3.1. Comparisons of the Growth Traits Between the G0 and G1 Generations

Growth performance is an important indicator during the artificial selection of *E. sinensis*. Larger individuals have more economic benefits than smaller ones, and they are preferred to meet market demand. The present study measured the growth traits of both the G0 and G1 generations, including their body weight, shell length, shell width, and third appendage length, and the difference in the growth performance between the G0 and G1 generations was compared (Table 1). The G1 generation showed better growth performance than the G0 generation. The increase in body weight, shell length, shell width, and third appendage length reached 8.3%, 7.9%, 9.6%, and 9.3%, respectively.

### 3.2. Summary of Whole-Genome Resequencing and Variation Calling

The present study generated 818.59G of raw reads by performing whole-genome sequencing on 40 *E. sinensis* individuals, with a Q30 of 91.63% and a GC content of 40.54%. A total of 793.34G of clean reads were obtained after removing the low-quality raw reads, with a Q30 of 93.54% and a GC content of 39.45%. A total of 99.19% clean reads were mapped to the reference genome of *E. sinensis*. In addition, the coverage ratios of ≥1× and ≥4× reached 83.97% and 72.34%, respectively, allowing the downstream analysis to be highly confident. 

A total of 372,448,393 high-quality SNPs were detected from the 40 *E. sinensis* individuals. According to Table 2, the number of SNPs, the number of heterozygous SNPs, the number of homozygous SNPs, and the ratio of TS/TV for each individual ranged from 8,912,691 to 10,134,760, from 5,006,688 to 6,130,454, from 3,891,116 to 4,086,108, and from 1.51 to 1.52, respectively, with an average of 9,331,209.83 and 5,334,483 SNPs and an average TS/TV ratio of 1.516. In addition, the average numbers of SNPs for start lost (SNPs result in the loss of a start codon), stop lost (SNPs result in the loss of a stop codon), stop gain (SNPs result in the gain of a stop codon), missense variant (missense variant in the exon regions), and synonymous variant (synonymous variant in the exon regions) were 162.8, 89.6, 375.2, 51,112.3, and 121,723.3, respectively. 

Insertions–deletions (InDels) were also identified in the present study based on the whole-genome resequencing data of the 40 *E. sinensis* individuals (Table 3). Among these 40 individuals, the insert number, delete number, heterozygous number, and homozygous number ranged from 989,852 to 1,196,941, from 1,173,576 to 1,398,171, from 1,209,485 to 1,560,106, and from 981,697 to 1,047,032, respectively, with an average of 1,060,698, 1,247,925, 1,296,086, and 1,012,538.

### 3.3. The LD Analysis, Phylogenetic Analysis, and PCA 

**Figure 2:**
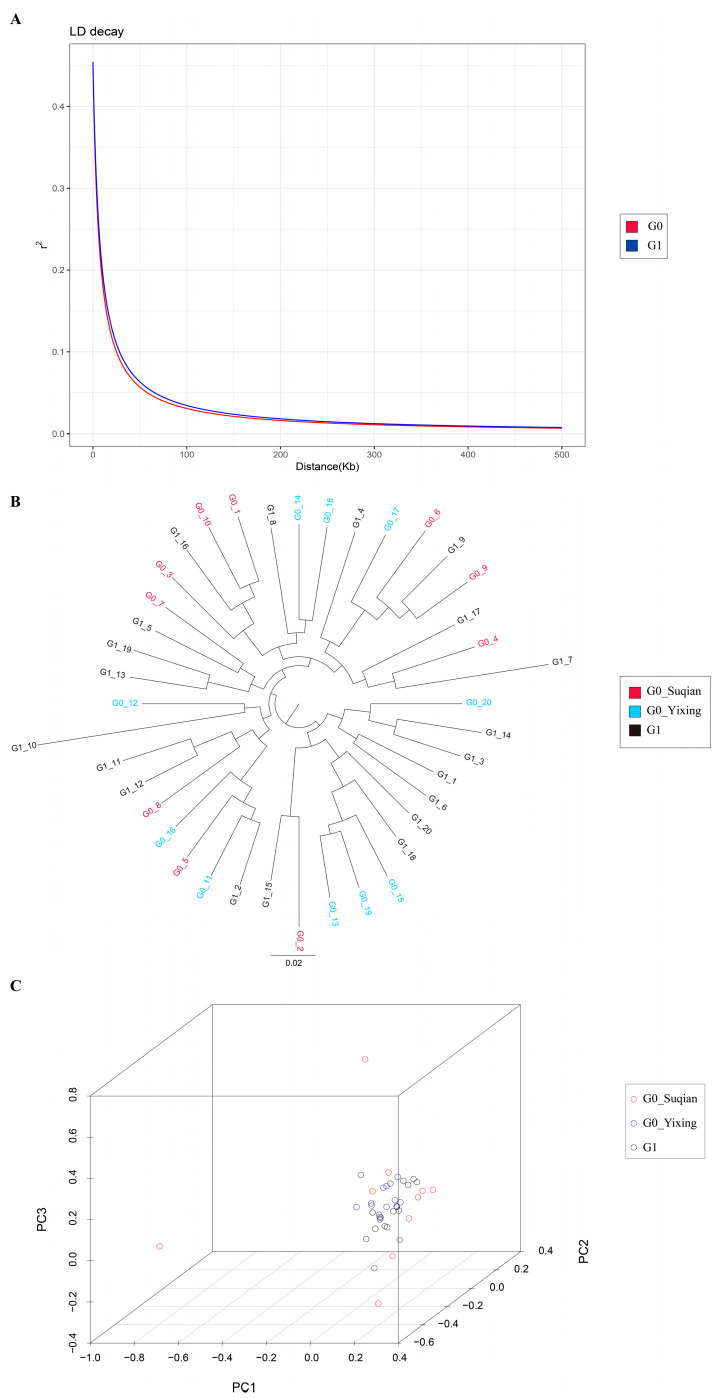
Linkage disequilibrium and population structure of 40 individuals. (**A**) Linkage disequilibrium analysis for the G0 and G1 generations. (**B**) Phylogenetic tree analysis for 40 individuals. (**C**) Principal component analysis for 40 individuals.

The decay of linkage disequilibrium (*LD*) in the G0 and G1 generations was analyzed by using the linkage disequilibrium measure (*r*^2^) method. The *LD* of G0 and PY weakened to 0.100054 and 0.109881, respectively, at a distance of 25 kb (Figure 2A). The phylogenetic tree and PCA revealed the genetic difference of 40 individuals from the G0 and G1 generations. The Yixing and Suqian populations did not reveal significant genetic differences (Figure 2B,C). This was supported by the *Fst* analysis, in which the *Fst* value between the Yixing and Suqian populations was only 0.007 (Table 4). Further analysis of the phylogenetic tree and PCA of 40 individuals from the G0 and G1 populations revealed that individuals from the G0 and G1 generations were not separately clustered (Figure 2B,C), which was consistent with the *Fst* analysis with an *Fst* value of 0.015 (Table 4). 

### 3.4. Genetic Diversity and Genetic Distance Analysis of the G0 and G1 Generations

The genetic diversity analysis of the G0 and G1 generations is shown in Table 4. The genetic diversity analysis revealed that the G0 and G1 generations did not show significant differences. The values of the observed heterozygosity, expected heterozygosity, *Pi*, *Fis*, *PIC*, and Shannon index in the G0 and G1 generations were almost the same. The values of the observed heterozygosity, expected heterozygosity, *Pi*, *Fis*, *PIC*, and Shannon index in the G0 generation were 0.368, 0.364, 0.375, 0.027, 0.289, and 0.540, respectively, compared with 0.379, 0.372, 0.383, 0.022, 0.294, and 0.549 in the G1 generation. 

## 4. Discussion

Artificial selection is still needed for the genetic improvement of *E. sinensis*. Our research group performed genetic improvement of *E. sinensis* for many years. The present study compared the growth performance between the G0 (pooled population of Suqian and Yixing crabs) and G1 generations (offspring of the G0 generation). Further, we analyzed the genetic diversity within the G0 and G1 generations and measured the genetic distance between them. The present study will provide valuable evidence for further genetic improvement in *E. sinensis*.

Body weight and body length are important economic traits that affect the economic value of *E. sinensis* [1]. Larger individuals are preferred due to market demand. In the present study, the G1 generation showed better growth performance than the G0 generation. Mass selection is an effective approach for the genetic improvement of aquatic animals, and it can be used to quickly improve certain target traits [49]. A previous study predicted that conventional breeding methods may lead to an 8–12% increase with every generation during the artificial selection of fish and shrimp [31]. 

The identification of LD patterns plays an essential role in the analysis of genetic diversity, reflecting the level of genetic diversity among different populations to some extent and showing similar results to those of the aforementioned indicators [50]. The phylogenetic tree and PCA revealed that individuals from the G0 generation (Yixing population and Suqian population) and the G1 generation did not show a distinct separation, indicating that crabs from these populations have a close genetic background. Further analysis revealed that the *Fst* values between the Yixing and Suqian populations and between the G0 and G1 generations were only 0.007 and 0.015, respectively. *Fst* refers to the genetic variation between populations or individuals that accounts for the total genetic variation [51]. The *Fst* analysis also provided evidence that the genetic background of the Yixing population, Suqian population, and G1 generation showed low genetic differentiation [52], which was consistent with previous studies showing that limited genetic differences were observed between populations from the Yangtze River [37,53].

Previous analyses of genetic diversity in aquatic animals were based on molecular markers, including random amplified polymorphic DNA (RAPD) [54,55], amplified length polymorphisms [56,57,58], and microsatellites [59,60,61]. In recent years, mitochondrial DNA (mtDNA) based on a D-loop has been applied to analyze the genetic diversity between different populations in an organism because of the rapid development of sequencing techniques [62,63,64]. However, WGS is a pivotal and useful method for identifying genetic variations and analyzing structural changes across different populations to enhance the understanding of molecular breeding in both animals and plants [65,66]. It allows for the precise correlation of phenotypic variations with genetic diversity among populations, especially in the context of modern breeding [67,68]. Previous studies have evaluated the germplasm resources and analyzed the genetic diversity and population structure of *E. sinensis* by using RAPD markers [35] and microsatellites [33,36,37]. The present study further explores the genetic diversity of *E. sinensis* by performing WGS. Previous studies identified that *Ho*, *He*, *Pi*, and *PIC* values were slightly lower in the selected population than in the wild population, indicating a slight loss of genetic diversity in the selective breeding progress of aquatic animals, including *Crassostrea gigas* [69], *Lates calcarifer* [70], *Salmo salar* [71], *Micropterus salmoides* [72], *Larimichthys crocea* [73], and *Ctenopharyngodon idella* [27]. A possible reason for this is that selecting favorable traits and genotypes through genetic breeding can lead to a loss of genetic diversity under the effects of selective sweep and background selection [74]. In the present study, the *Ho*, *He*, *Pi*, and *PIC* values remained stable between the G0 and G1 generations. To the best of our knowledge, a genetic diversity analysis has rarely been reported between different generations during the process of genetic breeding in *E. sinensis*. A possible reason for this is that we only performed the selective breeding progress for one generation; thus, no obvious decrease was observed between the G0 and G1 generations. In addition, a low genetic differentiation was reported in the selective process of aquatic animals, which was consistent with the present study. Heterozygosity and nucleotide diversity (*Pi*) are suitable indicators for evaluating the genetic diversity in a population or a species [75]. In the present study, the nucleotide diversity (*Pi*) in the G0 generation and G1 generation reached 0.375 and 0.383, respectively. The *PIC* values in the G0 generation and G1 generation were 0.289 and 0.294, respectively. All of these results indicated that high genetic diversities were observed in the G0 generation and G1 generation [76]. In addition, the *Ho* and *He* values in the G0 generation and G1 generation were very close, indicating a relatively stable genetic structure [25]. Inbreeding coefficients (*Fis*) play essential roles in predicting the inbreeding rate. In this study, the *Fis* values in both the G0 and G1 generations were close to 0, indicating an excess of heterozygosity in populations due to non-random mating [27].

## 5. Conclusions

The body weight, shell length, shell width, and third appendage length from the individuals of the G1 generation were dramatically increased compared with those of the G0 generation, indicating that the genetic breeding of *E. sinensis* through mass selection had progressed well. In addition, WGS generated 372,448,393 high-quality SNPs from 40 *E. sinensis* individuals. Phylogenetic tree analysis, PCA, and *Fst* analysis revealed that the Yixing and Suqian populations, along with the G1 generation, shared a closely related genetic background. This finding, derived from high-quality SNP data, suggests that genetic information was stably transmitted from the G0 generation to the G1 generation without the introduction of extraneous genetic material during breeding. Genetic diversity analysis revealed that the values of observed heterozygosity, expected heterozygosity, *Pi*, *Fis*, *PIC*, and Shannon index in the G0 and G1 generations were almost the same, indicating a stable genetic structure across these cohorts. In addition, the *Pi* and *PIC* values suggested high genetic diversities in the G0 and G1 generations. The present study evaluated the progress of genetic breeding of *E. sinensis* through WGS, promoting further genetic improvement of this species.

## Figures and Tables

**Table 1:** Measurement of the growth traits of the G0 and G1 generations.

	G0	G1
Body weight (g)	254.25 ± 43.09	275.35 ± 49.37 *
Shell length (cm)	9.56 ± 0.89	10.32 ± 1.23 *
Shell width (cm)	8.84 ± 0.81	9.69 ± 1.17 *
Third appendage length (cm)	16.78 ± 1.94	18.34 ± 2.35 *

Note: * indicates *p* < 0.05. Data are shown as the mean ± SD (standard deviation).

**Table 2:** Identification of SNPs through WGS.

	SNP Number	TS/TV	Heterozygous SNPs	Homozygous SNPs	Start Lost	Stop Lost	Stop Gain	Missense Variant	Synonymous Variant
G0_1	9,754,800	1.51	5,752,496	4,002,304	168	102	399	53,105	126,281
G0_2	9,189,324	1.52	5,169,226	4,020,098	166	97	361	51,296	121,659
G0_3	9,544,956	1.51	5,622,007	3,922,949	166	85	414	51,770	123,862
G0_4	9,451,226	1.51	5,463,120	3,988,106	160	98	356	51,610	121,732
G0_5	9,194,968	1.51	5,259,744	3,935,224	153	80	380	50,763	120,474
G0_6	9,280,159	1.52	5,239,791	4,040,368	172	90	388	51,370	121,525
G0_7	9,270,024	1.52	5,378,908	3,891,116	167	88	375	51,060	121,288
G0_8	8,912,691	1.52	5,006,688	3,906,003	163	88	348	49,671	118,080
G0_9	9,771,163	1.51	5,728,151	4,043,012	166	87	400	53,203	126,798
G0_10	10,134,760	1.51	6,130,454	4,004,306	168	107	415	54,529	129,120
G0_11	9,096,676	1.52	5,171,989	3,924,687	164	86	380	50,253	119,739
G0_12	9,215,241	1.52	5,270,094	3,945,147	171	98	364	50,849	120,967
G0_13	9,286,645	1.52	5,337,502	3,949,143	156	80	354	50,339	120,542
G0_14	9,176,043	1.52	5,185,922	3,990,121	150	90	386	50,754	121,462
G0_15	9,287,882	1.52	5,305,656	3,982,226	149	86	395	51,330	121,056
G0_16	9,205,017	1.52	5,303,025	3,901,992	155	98	359	51,188	120,982
G0_17	9,323,933	1.52	5,317,786	4,006,147	168	84	349	50,895	121,463
G0_18	9,236,822	1.52	5,338,032	3,898,790	167	87	392	51,543	122,196
G0_19	9,219,011	1.52	5,301,726	3,917,285	155	89	366	51,586	122,547
G0_20	9,237,297	1.52	5,307,690	3,929,607	168	92	382	51,788	122,125
G1_1	9,331,184	1.51	5,280,836	4,050,348	169	95	389	50,484	120,948
G1_2	9,050,275	1.51	5,136,972	3,913,303	166	89	391	49,949	118,785
G1_3	9,083,473	1.51	5,053,725	4,029,748	162	85	369	49,906	117,519
G1_4	9,410,135	1.52	5,487,990	3,922,145	169	88	380	52,006	124,606
G1_5	9,291,463	1.52	5,327,388	3,964,075	153	96	357	50,888	121,391
G1_6	9,357,440	1.52	5,417,257	3,940,183	161	89	344	51,020	122,967
G1_7	9,552,389	1.51	5,479,487	4,072,902	165	75	381	51,849	123,318
G1_8	9,018,329	1.51	5,042,342	3,975,987	167	81	351	49,494	118,921
G1_9	9,470,902	1.51	5,384,794	4,086,108	171	86	407	52,121	124,931
G1_10	9,414,264	1.51	5,353,963	4,060,301	168	83	367	51,113	121,095
G1_11	9,566,712	1.51	5,535,948	4,030,764	158	89	382	51,553	123,389
G1_12	8,980,803	1.52	5,058,556	3,922,247	160	87	362	49,273	118,895
G1_13	9,487,238	1.52	5,510,671	3,976,567	155	98	358	51,481	123,543
G1_14	9,335,793	1.51	5,263,304	4,072,489	157	103	383	51,011	120,802
G1_15	9,071,198	1.52	5,087,824	3,983,374	155	82	365	50,270	119,937
G1_16	9,232,880	1.52	5,158,971	4,073,909	164	91	373	50,893	120,452
G1_17	9,059,337	1.52	5,120,718	3,938,619	165	86	370	49,931	119,024
G1_18	9,660,963	1.51	5,658,768	4,00,2195	174	89	377	52,028	123,894
G1_19	8,995,646	1.51	5,072,810	3,922,836	150	96	383	49,231	118,826
G1_20	9,289,331	1.51	5,356,969	3,932,362	171	82	357	51,088	121,790
Average	9,331,209.83	1.516	5,334,483	3,976,727	162.8	89.6	375.2	51,112.3	121,723.3

**Table 3:** Identification of InDels through WGS.

	Insert Number	Delete Number	Heterozygosity Number	Homozygosity Number
G0-1	1,132,804	1,323,542	1,429,930	1,026,416
G0-2	1,024,615	1,212,128	1,220,646	1,016,097
G0-3	1,099,493	1,289,665	1,386,477	1,002,681
G0-4	1,082,365	1,272,755	1,337,657	1,017,463
G0-5	1,028,585	1,216,646	1,251,721	993,510
G0-6	1,052,100	1,239,119	1,259,166	1,032,053
G0-7	1,042,058	1,231,566	1,288,948	984,676
G0-8	989,852	1,173,576	1,181,731	981,697
G0-9	1,142,440	1,334,660	1,434,465	1,042,635
G0-10	1,196,941	1,398,171	1,560,106	1,035,006
G0-11	1,018,903	1,201,208	1,227,505	992,606
G0-12	1,033,663	1,224,262	1,258,677	999,248
G0-13	1,054,622	1,244,566	1,298,777	1,000,411
G0-14	1,031,480	1,216,363	1,237,760	1,010,083
G0-15	1062338	1,249,652	1,295,912	1,016,078
G0-16	1,031,929	1,223,291	1,267,028	988,192
G0-17	1,071,839	1,256,888	1,307,238	1,021,489
G0-18	1,037,292	1,225,798	1,273,253	989,837
G0-19	1,032,431	1,217,429	1,261,864	987,996
G0-20	1,033,738	1,222,643	1,264,236	992,145
G1-1	1,073,037	1,257,785	1,293,273	1,037,549
G1-2	1,011,695	1,197,173	1,223,109	985,759
G1-3	1,023,049	1,210,696	1,209,485	1,024,260
G1-4	1,078,355	1,267,127	1,346,951	998,531
G1-5	1,061,888	1,250,510	1,297,597	1,014,801
G1-6	1,074,536	1,261,356	1,329,519	1,006,373
G1-7	1,110,250	1,298,629	1,362,677	1,046,202
G1-8	1,018,560	1,198,678	1,209,576	1,007,662
G1-9	1,092,415	1,280,758	1,333,832	1,039,341
G1-10	1,084,350	1,272,979	1,318,102	1,039,227
G1-11	1,109,787	1,301,158	1,378,564	1,032,381
G1-12	1,017,467	1,191,887	1,216,603	992,751
G1-13	1,094,026	1,282,153	1,357,518	1,018,661
G1-14	1,072,343	1,257,805	1,283,182	1,046,966
G1-15	1,032,115	1,213,925	1,234,675	1,011,365
G1-16	1,058,302	1,240,648	1,251,918	1,047,032
G1-17	1,029,100	1,210,883	1,239,559	1,000,424
G1-18	1,112,482	1,307,360	1,394,861	1,024,981
G1-19	1,016,386	1,195,369	1,216,119	995,636
G1-20	1,058,288	1,246,212	1,303,215	1,001,285
Average	1,060,698	1,247,925	1,296,086	1,012,538

**Table 4:** Genetic diversity and genetic distance analysis.

Generation	Obs Het	Exp Het	*Pi*	*Fis*	*PIC*	Shannon Index	*Fst* YX vs. SQ	*Fst* G0 vs. G1
G0	0.368	0.364	0.375	0.027	0.289	0.540	0.007	0.015
G1	0.379	0.372	0.383	0.022	0.294	0.549		

Note: Obs Het indicates the observed heterozygosity. Exp Het indicates the expected heterozygosity. *Pi* indicates the nucleotide diversity. *Fis* indicates the inbreeding coefficient. *PIC* indicates the polymorphism information content. *Fst* indicates the genetic differentiation. YX indicates the Yixing population. SQ indicates the Suqian population.

## Data Availability

Publicly available datasets were analyzed in this study. The data have been submitted upon request.
